# The complete chloroplast genome of *Ophiopogon bodinieri* Levl. and its phylogenetic position

**DOI:** 10.1080/23802359.2021.1895000

**Published:** 2021-03-18

**Authors:** Xinchun Guo, Li He

**Affiliations:** a Key Laboratory of Poyang Lake Basin Agricultural Resource and Ecology of Jiangxi Province, College of Land Resource and Environment, Jiangxi Agricultural University, Nanchang, China; b College of Life Science, Jinggangshan University, Ji’an, China

**Keywords:** *Ophiopogon bodinieri*, chloroplast genome, phylogenetic

## Abstract

*Ophiopogon bodinieri* Levl. is an important turfgrass and ornamental cover plants which is widely used in urban garden construction in southern China. In this study, we sequenced the complete chloroplast genome of *O.bodinieri* on the Illumina HiSeq Platform. The chloroplast genome is 157,078 bp in length, with a typical quadripartite structure and consisting of a pair of inverted repeat (IR) regions (26,477 bp) separated by a large single copy (LSC) region (85,374 bp) and a small single copy (SSC) region (18,750 bp). It was predicted to contain a total of 132 genes, with an overall GC content of 38.63%. Phylogenetic analysis suggested *O.bodinieri* is closely relatedto *Goodyera velutina*, *Anoectochilus emeiensis* and *Ludisia discolor*.

*Ophiopogon bodinieri* Levl. Levl is an important turfgrass and ornamental cover plants which is widely used in urban garden construction in southern China (Liu et al. 2011). It is mainly distributed in tropical, subtropical and temperate regions of South and Southeast Asia (Wang and Yang 2015). It’s leaves are dark green, glossy, fine texture, and it has tense tillering ability, annual green period, high heat resistance and cold resistance (Xie et al. 2013). Despite extensive studies on the morphology, phytophysiology and biochemistry of *O.bodinieri*, the genetic information for this species remains quite limited. At present, there are few studies on the *O.bodinieri* genome. The plastome is valuable in plant systematics research due to its highly conserved structures, uniparental inheritance, and haploid nature (Fu et al. 2016). Plastome have also been smartly engineered to confer useful agronomic traits and/or serve as bioreactors (Jin and Daniell 2015). Here, we assembled and characterized the complete chloroplast genome sequence of *O.bodinieri* to provide information for the identification of Ophiopogon, as well as assist further phylogenetic study of Asparagaceae.

The plant material of *O.bodinieri* was collected from Jinggangshan University (N 27°06′ 45.46″, E 115°01′55.84″'), Ji’an, Jiangxi Province,China. The voucher specimen of *O.bodinieri* has been kept in Key Laboratory of Ecological Environment and Resource Utilization, Jinggangshan University. The specimen accession number is JGSU20190706.The total genomic DNA was extracted from fresh leaves using the modified CTAB method (Doyle and Doyle 1987). The DNA library was prepared with a TruSeq DNA Sample Prep Kit (Illumina, USA) according to the instructions of the manufacturer. A genomic DNA library with an insert size of 400 bp was constructed and then sequenced by an Illumina HiSeq 2500 system. Approximately 4.6 GB of clean data were yielded and then *de novo* assembled using CLC genome assembler program (ver.4.06 beta, CLC Inc, Aarhus, Denmark) as previously described (Kim et al. 2015), and filtered sequences were assembled using the program SPAdes assembler v3.10.1 (Bankevich et al. 2012). Finally, CpGAVAS2 was used to annotate the gene structure (Shi et al. 2019). The obtained sequence was submitted to GenBank under the accession number MT937174.

The complete chloroplast genome of *O.bodinieri* is circular molecular structure of 157,078 bp in length, consisting of two inverted repeats (IR) regions of 26,477 bp, separated by large single-copy (LSC) and small single-copy (SSC) regions of 85,374 bp and 18,750 bp, respectively. The overall GC content of the chloroplast genome was 37.59%, while the corresponding values of the LSC, SSC, and IR regions were 35.62%, 31.21%, and 43.02%, respectively. The chloroplast genome comprises 132 genes, including 86 protein-coding genes, 38 tRNA genes, and 8 rRNA genes. 18 genes occurred in the IR region have two copies, including 6 protein-coding genes(*rps19*, *rpl2*, *rpl23*, *ycf2*, *rps7* and *ndhB*), 8 tRNA genes(*trnH-GUG*, *trnM-CAU*, *trnL-CAA*, *trnV-GAC*, *trnI-GAU*, *trnA-UGC*, *trnR-ACG*, *trnN-GUU*), and 4 rRNA genes(*rrn16*, *rrn23*, *rrn4.5*, *rrn5*). There are 111 unique genes, among which 16 genes contained one intron, and two genes (*clpP* and *ycf3*) contained two introns.

To investigate the relationship of *O.bodinieri*, the chloroplast genomes of *O.bodinieri* and 15 other species were aligned using MAFFT ver. 7.307 (Katoh and Standley 2013), *Oncidium hybrid cultivar* and *Erycina pusilla* were used as outgroup. A phylogenetic tree (Figure 1) was constructed with the maximum likelihood method using RAxML (Stamatakis 2014). The result of the phylogenetic analysis revealed that *O.bodinieri* is not monophyletic. The *O.bodinieri* is closely related to *Goodyera velutina*, *Anoectochilus emeiensis* and *Ludisia discolor*. The completion and characterization of the complete plastid genome sequence in this study provided helpful molecular resource for evolutionary studies of the valuable turfgrass *O.bodinieri* and its allies in the future.

**Figure 1.**
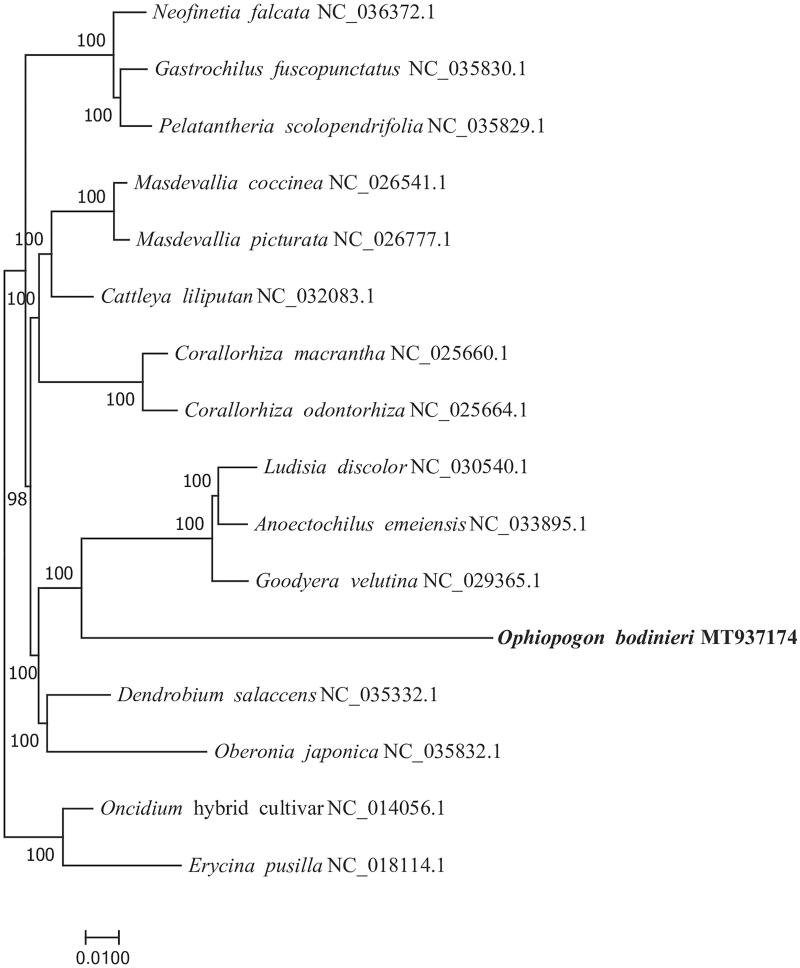
Phylogenetic tree inferred by maximum-likelihood (ML) method based on the complete chloroplast genomes of 16 representative species. Numbers near the nodes mean bootstrap support value.

## Data Availability

The genome sequence data that support the findings of this study are openly available in GenBank of NCBI at [https://www.ncbi.nlm.nih.gov] (https://www.ncbi.nlm.nih.gov/) under the accession no MT937174. The associated BioProject, SRA, and Bio-Sample numbers are PRJN668235, SRR12809560, and SAMN16401694 respectively.
